# Serum Level of Vitamin D and Febrile Seizure? A Clinical Study

**Published:** 2020

**Authors:** Farhad HEYDARIAN, Elham BAKHTIARI, Hasan GOLMAKANI, Neda FAKHR GHASEMI, Mohammad HEIDARIAN

**Affiliations:** 1 Research Center for Patients Safety, Mashhad University of Medical Sciences, Mashhad, Iran; 2 Eye Research Center, Mashhad University of Medical Sciences, Mashhad, Iran; 3 Department of Pediatrics, Faculty of Medicine, Mashhad University of Medical Sciences, Mashhad, Iran; 4 General physician, Mashhad University of Medical Sciences, Mashhad, Iran; 5 Biological sciences student, California state university, East Bay, Hayward, California

**Keywords:** Vitamin D, febrile seizure, Child

## Abstract

**Objective:**

To evaluate the serum level of vitamin D in children aged six to 60 months with febrile seizure and febrile children without the seizure.

**Materials & Methods:**

Febrile children aged six to 60 months with or without seizure were studied. Demographic characteristics, serum level of vitamin D, and other laboratory findings were recorded.

**Results:**

Among the 104 children, 51 patients had fever without a seizure and 53 patients had a febrile seizure. The mean subjects’ age was significantly more in the febrile seizure group compared to the without seizure group (16.26 ± 11.87 versus 26.36 ± 14.11 months, p = 0.001). The mean serum level of vitamin D in the with and without seizure groups was 41.92 ± 22.42 and 48.41 ± 15.25 microgram per deciliter, respectively (p = 0.08). There was no significant correlation between serum level of vitamin D and seizure occurrence (p = 0.07). The mean serum sodium and potassium levels, and platelet count were significantly lower in the febrile seizure group compared to the without seizure group (p < 0.05). There were no significant differences between the two groups regarding hemoglobin, blood sugar, creatinine, blood urea nitrogen, calcium, alkaline phosphatase levels, and white blood cell count (p > 0.05).

**Conclusion:**

The serum level of vitamin D in febrile children with or without seizure was normal. The serum level of vitamin D was lower in patients with the seizure but not statistically significant. More clinical studies are needed to evaluate the relationship between febrile seizure and the serum level of vitamin D.

## Introduction

Febrile seizure (FS) is one of the common childhood neurologic diseases that involves three to five percent of children (1, 2). FS is recurrent in 30% of patients (3, 4). Despite many proposed mechanisms, its exact pathogenesis is unknown. Genetic and environmental factors are assumed to be involved in its mechanism (5). Seizures can be the outcome of several conditions stimulating the central nervous system (CNS) such as fever, electrolyte disturbances, some infections, head trauma, etc. (6, 7). 

Vitamin D deficiency is a prevalent public health problem throughout the world, particularly in developing countries. Vitamin D deficiency is more common during the rapid growth stages, such as infancy and adolescence (8). The prevalence of vitamin D deficiency and insufficiency in Iranian children is 16.1% and 25.2%, respectively (9). Vitamin D receptors exist throughout the CNS. Vitamin D plays an essential role in the function of neurotransmission in the CNS of children. Some studies showed the relationship between low serum level of vitamin and epilepsy in children (10), but the relationship between FS and vitamin D deficiency was recognized recently. The clinical studies evaluating the relationship between vitamin D deficiency and the febrile seizure in children are limited in the literature. There are only a few case report studies (11-15).

The present study was designed to evaluate the relationship between serum level of vitamin D and FS in the children aged six to 60 months. 

## Materials & Methods

This clinical study was performed in Ghaem Hospital, Mashhad University of Medical Sciences (Mashhad, Iran) from December 2016 to April 2018. The study protocol was approved by the Ethics Committee of Mashhad University of Medical Sciences (code: IR.MUMS.fm.REC.1395.505). The study participants were 104 admitted febrile children, aged six to 60 months with or without the first episode of seizure. The diagnosis of seizure was according to the symptoms and the findings of clinical examination. Written informed consent was obtained from the parents before the beginning of the study. A blood sample was taken from all included patients. The serum levels of vitamin D, hemoglobin (Hb), blood sugar (BS), creatinine (Cr), blood urea nitrogen (BUN), calcium (Ca), potassium (K), sodium (Na), alkaline phosphatase (ALP), phosphorus (P), and platelet and white blood cells (WBC) counts were measured. 

 Patients with a history of acute or chronic renal diseases, meningitis, febrile convulsion or epilepsy, head trauma, encephalitis, and brain hemorrhage were excluded. 

**Sample size**

Sample size study populations of 104 patients were considered appropriate to achieve a reasonable statistical analysis. 

**Analysis**

Statistical analysis was performed using SPSS windows program version 16 (SPSS Institute, Inc., Chicago, IL, USA). All quantitative variables were described as mean ± standard deviation (SD) and compared between the two groups by independent samples t-test or its nonparametric equivalent. The qualitative variables were compared between the two groups using Chi-square test. P-values less than 0.05 were considered statistically significant.

## Results

***Demographic characteristics***

Among the 104 included children, 51 patients had fever without a seizure and 53 patients had a febrile seizure. In without seizure group, 24 cases (47.1%) were female and 27 cases (52.9%) were male with the mean age of 16.26 ± 11.87 months. In the FS group, 26 cases (49.1%) were female and 27 cases (50.9%) were male with the mean age of 26.36 ± 14.11 months. There was no significant difference between the two groups regarding the subjects’ gender (p = 0.999). The mean subjects’ age was significantly more in the febrile seizure group compared to the without seizure group (p = 0.001, Table 1). 

The mean body temperature was 38.85 ± 0.55 and 38.38 ± 1.97 ^0^C in with and without seizure groups, respectively (p = 0.13).

There were also no significant differences regarding the existence of underlying disease (p = 0.14), type of parturition (p = 0.999), and breastfeeding (p = 0.75) between the two groups.

***Seizure characteristics***

Fifthly-three patients had a febrile seizure. Forty-seven patients (88.7%) had one episode of seizure. Only three patients (5.7%) had a positive family history of seizure. The type of seizure was generalized in 39 patients (73.9%).

***Laboratory data***

The mean of vitamin D serum level in with and without seizure groups was 41.92 ± 22.42 and 48.41 ± 15.25 microgram/deciliter (µg/dL), respectively (p = 0.08). There was no significant correlation between serum level of vitamin D and seizure occurrence (p = 0.07). According to the logistic regression, the odds ratio for seizure in febrile patients with the serum level of vitamin D less than 30 µg/dL was 2 but not statistically significant (OR = 2, p = 0.13). Among the 104 children, there was no significant difference in the serum level of vitamin D in children aged less than one year and children aged more than one year (48.19 ± 17.14 versus 43.6 ± 21.23 µg/dL, p = 0.31). Also, in patients with FS, there was no significant difference in serum level of vitamin D between children aged less than one year and children aged more than one year (45.24 ± 21.02 versus 41.54 ± 23.56 µg/dL, p = 0.58). The serum level of vitamin D in febrile patients aged less than one year with or without seizure was 45.24 ± 21.02 and 49.75 ± 15 µg/dL, respectively (p = 0.51). The mean serum level of vitamin D in febrile patients aged more than one year with or without seizure was 41.54 ± 23.56 and 47.93 ± 14.83 µg/dL, respectively (p = 0.28).

The mean platelet count was 260.07 ± 129.88 and 324.93 ± 116.51 cell/µL in with and without seizure groups, respectively. The difference between the two groups regarding mean platelet count was statistically significant (p = 0.015). 

The mean serum potassium level in the with and without seizure groups was 4.08 ± 0.39 and 4.33 ± 0.42 mmol/L, respectively. The difference between the two groups regarding the serum potassium level was statistically significant (p = 0.007). 

The serum sodium level in patients with and without seizure was 137.4 ± 2.44 and 141.12 ± 7.36 meq/L, respectively. The difference between the two groups in serum sodium level was also statistically significant (p = 0.002). 

The serum phosphorus level was 4.09 ± 0.74 and 4.59 ± 0.71 mg/dL in patients with and without seizure, respectively (p = 0.058). Differences between the two groups regarding serum levels of Hb, WBC, BS, Cr, BUN, Ca, and ALP was not statistically significant (p > 0.05, Table 2).

**Table1:** Demographic characteristics of 104 febrile patients with and without seizure

**P value**	**Fever without seizure**	**Febrile seizure**	
	51	53	**Number of subjects**
0.001*	16.26 ± 11.87	26.36 ± 14.11	**Age (month)**
0.999	27 (52.9%) 24 (47.1%)	27 (50.9%) 26 (49.1%)	**Gender** **Male** **Female**

* Statistically significant according to the independent samples *t* test.

**Table2:** Laboratory data of 104 febrile patients with and without seizure

**P value**	**Fever without seizure** **(n=51)**	**Febrile seizure** **(n=53)**	
0.08	48.41 ± 15.25	41.92 ± 22.42	**Vitamin D (microgram/dL)**
0.015*	324.93 ± 116.51	260.07 ± 129.88	**Platelet (cells/microL)**
0.007*	4.33 ± 0.42	4.08 ± 0.39	**Potassium (mmol/L)**
0.002*	141.12 ± 7.36	137.4 ± 2.44	**Sodium (meq/L)**
0.058	4.59 ± 0.71	4.09 ± 0.74	**Phosphorus (mg/dL)**
0.90	10.95 ± 1.54	10.92 ± 1.06	**Hb (gram/dL)**
0.42	11.28 ± 4.49	10.46 ± 5.07	**WBC (cell/microL)**
0.27	105.84 ± 33	113.23 ± 28.01	**BS (mg/dL)**
0.305	0.51±0.22	0.47±0.08	**Cr (mg/dL)**
0.07	26.87 ± 13.99	22.05 ± 9.8	**BUN (mg/dL)**
0.38	9.79 ± 0.79	9.6 ± 0.61	**Ca (mg/dL)**
0.84	519.36 ± 170.6	530.92 ± 181.39	**ALP (unit/L)**

* Statistically significant according to the independent samples *t* test.

Abbreviations: Deciliter (dL); liter (L); millimole (mmol); milliequivalent (meq); milligram (mg)

## Discussion

The serum vitamin D level of one-hundred four febrile patients with or without seizure was studied. Although the serum level of vitamin D in patients with seizure was lower than the patients without a seizure (41.92 ± 22.42 versus 48.41 ± 15.25 µg/dL), this difference between the two groups was not statistically significant. Although it was not statistically significant, the serum level of vitamin D was higher in patients under one year old than the others. It may be due to the administration of vitamin D supplements in these patients. Vitamin D receptors broadly spread in the brain that affecting calcemic and non-calcemic actions (16). The differences regarding serum levels of platelet, sodium, and potassium were significant between groups, but Hb, WBC, BS, Cr, BUN, Ca, and ALP was not. 

The anticonvulsant nature of vitamin D initially was reported in 1974 (17). Vitamin D receptors exist widely in the brain, and vitamin D can act as a neurotransmitter. It also may enhance the effect of other neuroprotectin agents. It has been suggested that some metabolic changes may occur during febrile disease (16). To the best of our knowledge, there is no clinical study evaluating and comparing the serum level of vitamin D in febrile children with and without seizures. 

In the present study, the serum level of vitamin D in both groups of with and without seizure was within the normal range, interestingly. The mean serum level of vitamin D in patients with seizures was lower than the patients without a seizure. In other reports, the low serum level of vitamin D in patients with seizures was reported frequently (6, 11, 12, 14). It may be to some extent due to differences in the design of the study, dietary habits, and geographic situation. In a recent study in Iran on 40 children with seizures, it has been reported that only 20% of them had a sufficient serum vitamin D level (6). In another study in Iran, the prevalence of insufficient vitamin D was 46.6% (18). 

In our study, the serum level of sodium was significantly lower in patients with seizures. It may be due to inappropriate secretion of anti-diuretic hormone (ADH). Also, the potassium level was lower in the case group. The actual reason is not detected yet. 

## In Conclusion,

Despite the normal level of vitamin D in febrile children with and without a seizure, the mean serum vitamin D level was lower in patients with seizures. More clinical studies are needed to evaluate the relationship between febrile seizure and vitamin D deficiency among the children.
